# Care-Seeking Pattern for Diarrhea among Children under 36 Months Old in Rural Western China

**DOI:** 10.1371/journal.pone.0043103

**Published:** 2012-08-17

**Authors:** Wenlong Gao, Shaonong Dang, Hong Yan, Duolao Wang

**Affiliations:** 1 Department of Epidemiology and Health Statistics, School of Public Health, College of Medicine, Xi'an Jiaotong University, Xi'an, Shaanxi, People's Republic of China; 2 Department of Medical Statistics, London School of Hygiene and Tropical Medicine, London, United Kingdom; Vanderbilt University, United States of America

## Abstract

**Objective:**

To explore the caretakers' care-seeking pattern and its determinants among children under 36 months old with diarrhea in rural western China.

**Methods:**

The data of 14112 households was collected in 45 counties of 10 provinces of western China from June to August 2005. A generalized estimated equation (GEE) linear model was used to identify the determinants of the care-seeking.

**Results:**

Village-level and township-level care were sought for childhood diarrhea by 67.02% of the caretakers. GEE model analysis shows that compared with the caretakers of the children delivered at county-level or above hospitals, those of the children delivered at home seldom sought a higher level care (−0.23, 95%CI: −0.45,−0.01, p = 0.040); that the age of the children was negatively associated with seeking a higher level care (12 vs 36 months: 0.35, 95%CI: 0.16,0.55, p<0.001; 24 vs 36 months: 0.26, 95%CI: 0.08,0.44, p = 0.004); that the more danger signs of diarrhea the caretakers recognized, the higher level care they sought for their children with diarrhea (0.04, 95%CI: 0.00,0.07, p = 0.037); that the children with breastfeeding were given a higher level care than those without (0.15, 95%CI: 0.01,0.28, p = 0.035); that the mothers with a higher education sought the higher level care than those with only primary education (0.29, 95%CI: 0.03,0.56, p = 0.032); and that the farther the villages where these caretakers lived were from their townships, the lower level care for their children with diarrhea they sought (−0.09, 95%CI: −0.18,−0.01, p = 0.039).

**Conclusion:**

Village-level and township-level care were sought for childhood diarrhea by most of the caretakers. Birth settings, the distance from village to township, maternal education, caretakers' awareness of the danger signs of diarrhea, breastfeeding status and age of children affected the care-seeking. These findings may have some implications for the improvement of health care services and care-seeking intervention against childhood diarrhea in rural western China.

## Introduction

Diarrhea remains the second most common cause of childhood mortality all over the world. [1]–[4]Globally, nearly one in five child deaths- about 1.5 million each year- is due to diarrhea. [1] In China annually, 40,000 children die of diarrhea and incidence is the highest in the first two years of life. [1]

Although most of the episodes in childhood diarrhea are mild, severe cases can lead to significant fluid loss and dehydration. [1] Dehydration is the main immediate cause of death from acute diarrhea. [5] Therefore, it is crucial that caregivers replace the fluids in a timely manner and seek appropriate care when even mild dehydration symptoms of diarrhea appear in a child. For example, a report from Mongolia showed that nearly 20% of the all-cause deaths in children under 5 years old is a result of not seeking immediate medical care and services. [6] Nevertheless, improving families' care seeking behavior could contribute significantly to reducing child mortality in developing countries. [7] Many studies of childhood diseases have also showed that early appropriate and prompt care are essential to reduce adverse outcomes. [8]–[10]

In rural China, village clinics, township hospitals and county hospitals form a three-tier health service system. Village clinics provide basic acute and preventative care, township hospitals public health services, ambulatory and basic inpatient care, and county hospitals specialized outpatient and inpatient care. [11] In the three-tier system, county hospitals provide the highest professional care, township hospitals the second, and village clinics the lowest. The higher level services are mainly funded by government.

In China, no study has been conducted of care-seeking behaviors for diarrhea among children under 36 months old. Our study intended to assess the caretakers' care-seeking behavior in this group of children with an aim to help policy makers to make informed decisions about the resource and infrastructural allocation by identifying the care-seeking patterns and understanding the likely influences on a person's behavior. [12] Additionally, health interview surveys can be relied on for more accurate information about morbidity and treatment patterns than those medical records. [13] Meanwhile, health educators can also obtain some insights of care-seeking behaviors for diarrhea among children under 36 months old in rural western China.

## Methods

### Ethics Statement

The study was reviewed and approved by the Ethics Committee of Medicine College of Xi'an Jiaotong University and written informed consent had been obtained from all the study participants.

### Setting and study population

The study used the data collected from the rural primary health care survey conducted in 45 counties of 10 provinces (Xinjiang, Inner Mongolia, Qinghai, Gansu, Ningxia, Sichuan, Chongqing, Guizhou, Jiangxi and Guangxi) of rural western China from June to August 2005. All the surveyed counties were directly determined by the Chinese Ministry of Health and the United Nations Children's Fund rather than sampled randomly. After a multi-stage probability-proportional-to-size sampling (PPS) method was adopted, [14] five townships were sampled from each county and then four villages were selected in each sampled township randomly. In each selected village, 16 households with children under 36 months old were extracted with a completely random sampling method. Health personnel of each village needed first to list a sheet of all households with children under 36 months old and then the interviewers could draw 16 households with the method of drawing lots after giving a unique number to each household. If a village had more than 16 households, 16 households were selected randomly; if a village had less than 16 households, all the households were selected and the rest were made up of the neighboring villages. Finally, only one child was selected randomly in every selected household and his/her caretaker was interviewed.

### Data collection

In the survey, all questionnaires including family questionnaire, village clinic questionnaire, township hospital questionnaire and county health questionnaire were designed by Chinese Ministry of Health and the College of Medicine of Xi'an Jiaotong University and the latter was responsible for conducting the survey. The sample for this study was from the family questionnaire and village clinic questionnaire. Family primary data was collected from the caretakers with the help of the pre-coded structured family questionnaire. After signing the informed consent form, all participants were interviewed face-to-face with the unified family questionnaire. The family questionnaire includes 5 parts: 1) general information of the family; 2) health care, feeding and the occurrence of the diseases (childhood cold and diarrhea); 3) maternal health care and pregnancy care; 4) anthropometrics and 5) cooperative medical treatment. There were a total of 98 questions in the family questionnaire. When collecting the information on the occurrence of diarrhea to a child, the interviewer asked the caretaker whether the child had had some symptoms such as watery or rice watery stools in the previous 2 weeks. If so, the interviewer continued to ask how often such symptoms appeared in the proceeding 24 hours. If the symptoms appeared 3 or more times, the child was identified as having a diarrhea. Then, the interviewer asked the information about the care-seeking, care settings, the recognition of the 7 dangerous symptoms of diarrhea (frequent watery stools in proceeding one or two hours, blood in stools, repeated vomiting, high fever, extreme thirst, no desire to drink and refusal to eat), oral vitamin A use in the previous year and receiving educational material about childhood diseases from health personnel to the caretakers of children with diarrhea. The recognition on the dangerous symptoms of diarrhea was based on a multiple choice question including 7 items: Do you think which of the following dangerous symptoms could make you send your child to see a doctor? To obtain an accurate response, investigators must explain the exact meaning of every item to interviewees. The information of birth setting was from the questionnaire about maternal health care. Each interview lasted about 30 minutes. To ensure the availability of data, each questionnaire had been reviewed by the leader of the investigation team and its appropriateness had been carefully verified before it was accepted. Village-level information was collected from the village health personnel with a village clinic questionnaire. The village clinic questionnaire consisted of 44 questions including the distance from village to township and to county, and the number of retail pharmacies.

### Main study variables

Care-seeking was considered as a unique outcome variable in the study. According to the places of the care in the last diarrheal episode during the previous two weeks, we identified four categories of care-seeking: home-based care-seeking (HBC), village-level care-seeking (VLC), township-level care-seeking (TLC) and county-level-or-above care-seeking (CLC). In order to facilitate the analysis, no-care (NC) was also included as a special kind of care-seeking. It meant that the caretakers had not taken any special care or treatment measures against diarrhea, such as increasing the frequency of feeding, increasing fluid intake, oral dehydration salt therapy or medical care and so on in the diarrhea episode. HBC indicated the caretakers took those special care or treatment measures against diarrhea at home. The delivery was also classified into four categories in accordance with the birth sites: home-based delivery (HBD), village-level delivery (VLD), township-level delivery (TLD) and county-level-or-above delivery (CLD). The study also took into account the number of the 7 dangerous symptoms recognized by caretakers of diarrhea which reflected the caretakers' capacity of deciding whether to seek promptly medical care for their children with diarrhea. Due to the lack of income data of each household, the socioeconomic status of the household was assessed by means of the Demographic and Health Survey wealth index. [15] After the principal component analysis of the five variables representing the family economic level (type of vehicle, water supply, income resource, texture of pot and type of television), according to the tertiles of the first principal component, the socioeconomic status of the families was classified into three categories: poor, medium and rich. Han ethnicity accounts for 92% of total population in China. Based on empirical studies and topic analysis, a total of 19 independent variables was included in this study: 4 multi-categorical variables (the province, the delivery mode, the socioeconomic status of the families and age of children), 4 continuous variables (recognition of danger signs of diarrhea, maternal age, distance from village to township and distance from village to county by 10 kilometers) and 11 dichotomous variables (receiving educational materials about childhood diseases, gender of children, maternal education, main caretaker, ethnicity, family size, pre-school-aged child size, the number of retail pharmacies in village, breastfeeding status, habit of drinking water and oral vitamin A in the previous year). For a quantitative predictor, some transformations such as quadratic, log arithmetic or square root were tested to find the best character of all independent variables.

### Data analysis

The data obtained from the questionnaires was entered in Epidata 3.1 by double entry and later analyzed with SPSS version17 (SPSS Inc, Chicago, IL, USA). The level of the significance of analysis was set at 0.05. A nonparametric test of Kruskal-Wallis H was employed to compare the overall proportion and one of Nemenyi was used to make the pairwise comparisons among the proportions. Nineteen study variables were together entered into a generalized estimated equation (GEE) linear model to identify the predictors of care-seeking (0 for NC; 1 for HBC; 2 for VLC; 3 for TLC; and 4 for CLC) while controlling for possible correlation in the care-seeking among the same village. The equation of GEE linear model can be expressed as follows:[16], [17]

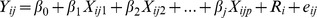
()Here, 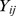
is the care-seeking level of the *j*-th subject of the *i*-th village (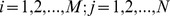). 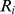
is the working correlated matrix and 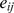 is the error term. The coefficient 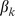 reflected the magnitude and direction of effect of *k*-th independent variable 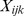 on the level of the care-seeking (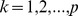). GEE model estimates the average response over the population (“population-averaged” effects) rather than the regression parameters that would enable prediction of the effect of changing one or more covariates on a given individual.

## Results

### Diarrhea prevalence and sample characteristics

**Figure 1:**
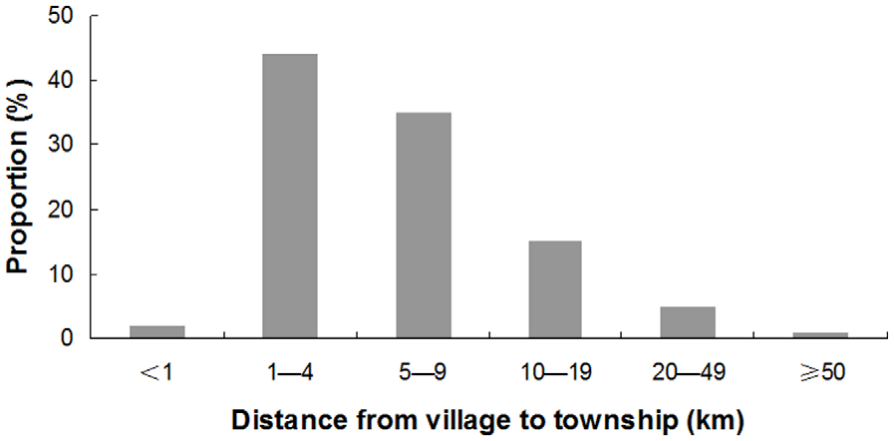
The distribution of distance from village to township.

**Figure 2:**
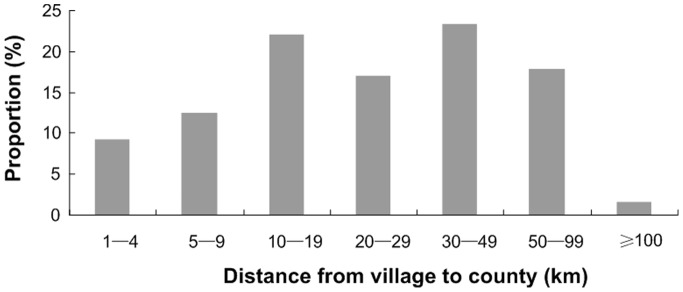
The distribution of distance from village to county.

Totally, 894 villages were sampled out of 225 townships and 14112 households were involved. Table 1 shows the diarrhea prevalence and care-seeking pattern for diarrhea among children under 36 months old in 10 provinces of rural western China. A total of 14112 caretakers were investigated and 1040 children living in 537 villages of 204 townships were found to have suffered from at least one diarrheal episode in the previous two weeks. The two-week prevalence rate of diarrhea in children under 36 months old was 7.37%. Of the ten provinces, the prevalence rate in Xinjiang was 14.53%, ranking the highest, and that in Chongqing, 2.33%, was the lowest. In the 537 villages, 88.07% had no retail pharmacies. The distance from these villages to their townships was 6.78 km (0–115 km Standard deviation (SD) = 7.57 km) and that from these villages to their counties was 29.86 km (1–147 km SD = 23.35 km) on average. Figure 1 and Figure 2 displayed the distribution of the distance from village to township and of that from village to county respectively.

**Table 1:** Diarrhea prevalence and care-seeking pattern for diarrhea among children under 36 months old in 10 provinces of rural western China.

Province	Number of households(n)	Households with children with diarrhea in previous two weeks (%)	aNC (%)	HBC (%)	Health care sectors		
					VLC (%)	TLC (%)	CLC (%)
Gansu	634	27(4.26)	3.70	3.70	66.67	18.52	7.41
Guangxi	1586	58(3.66)	5.17	12.07	46.55	18.97	17.24
Guizhou	1265	116(9.17)	7.76	6.03	59.48	21.55	5.17
Jiangxi	1567	74(4.72)	12.16	4.05	50.00	21.62	12.16
Inner Mongolia	1217	65(5.34)	4.62	21.54	24.62	27.69	21.54
Ningxia	1264	79(6.25)	10.26	6.41	34.62	33.33	15.38
Qinghai	1589	119(7.49)	10.00	11.67	50.83	17.50	10.00
Sichuan	1577	158(10.02)	6.33	10.13	39.24	34.18	10.13
Xinjiang	2168	315(14.53)	10.79	25.40	28.57	30.48	4.76
Chongqing	1245	29(2.33)	6.90	20.69	20.69	41.38	10.34
All	14112	1040(7.37)	8.75	14.71	39.71	27.31	9.52

a NC, no care; HBC, home-based care-seeking; VLC, village-level care-seeking; TLC, township-level care-seeking, CLC, county-level-or-above care-seeking.

**Figure 3:**
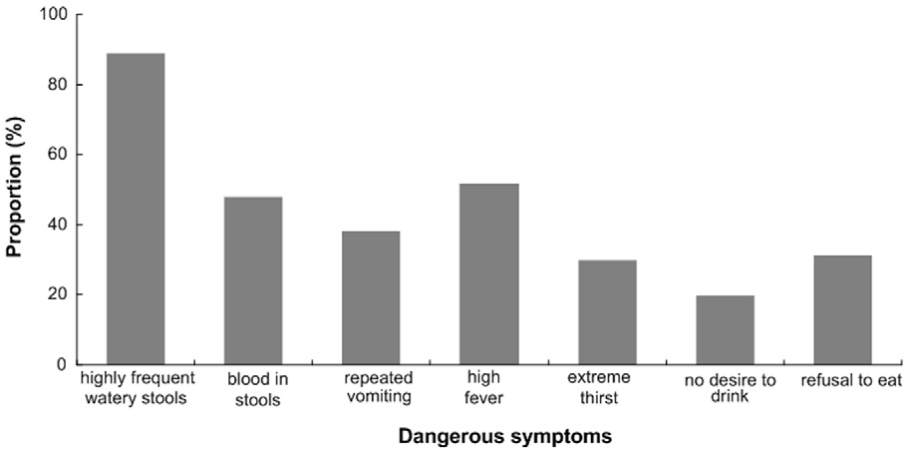
The proportions of caretakers' dangerous symptoms recognized of diarrhea among children under 36 months old.

Table 2 shows the characteristics of the families, caretakers and children with diarrhea and village-level information. Of the studied families, slightly less than a half had more than four members and more than a half (56.15%) had only one pre-school-aged child, most (80.67%) drank boiled water often and slightly more than a half had a poor socioeconomic status. Of the caretakers, most (83.94%) were mothers of the children, slightly more than one half (52.98%) were of Han ethnicity. Of the mothers of children with diarrhea, nearly one third (32.88%) was less than 25 years old and their average age was 27 years. More than 90% of these mothers never went to a senior high or higher-level school. Only 8.65% of the caretakers were able to recognize all of seven dangerous symptoms of diarrhea, still 2.40% could not recognize any one and less than one-third could recognize more than 3 dangerous symptoms. Figure 3 showed the proportions of 7 dangerous symptoms of diarrhea recognized by the caretakers. More than three-quarters (79.13%) of them acquired some educational materials about childhood diseases from health personnel. Of the children with diarrhea, 615 were boys and 425 girls. Most of them were less than 24 months old and their average age was 15 months. Over half (51.35%) of them were still being breastfed and also more than half (56.06%) had swallowed oral vitamin A in the previous year. Over three-quarters (78.07%) of them were delivered at township or higher level hospitals, less than one fifth at home, and less than 4% in village clinics.

**Table 2:** Characteristics of families, caretakers and diarrhea children, and village-level information.

Characteristics	n	%
**Family**		
Family size		
<5	543	52.21
≥5	497	47.79
Pre-school-aged children size		
1	584	56.15
>1	456	43.85
The habit of drinking water		
Boiled water often	839	80.67
Not or occasional boiled water	201	19.33
Socioeconomic status of the households		
Rich	275	26.44
Medium	209	20.10
Poor	556	53.46
**Caretakers**		
Main caretaker		
Mother	873	83.94
Others	167	16.06
Ethnicity		
Han	551	52.98
Minority	489	47.02
aAge of mothers(mean SD) [range]	26.94, 4.79 [18–44]	
Maternal education (year)		
0–9	964	92.69
>9	76	7.31
Receiving educational materials about childhood diseases		
Receiving	823	79.13
Not receiving	217	20.87
bThe number of recognizing danger signs(mean, SD)	2.92,1.85	
**Children with diarrhea**		
Age of children		
0–12 months	509	48.94
13–24 months	360	34.62
25–36 months	171	16.44
cThe delivery		
HBD	194	18.65
VLD	34	3.26
TLD	433	41.63
CLD	379	36.44
Gender of children		
Boy	615	59.13
Girl	425	40.87
Breastfeeding when surveyed		
Being breastfed	534	51.35
Not breastfed	506	48.65
Oral vitamin A in the previous year		
Use	583	56.06
Not use	457	43.94
d **Village-level information**		
The number of retail pharmacies in the village		
Zero	465	88.07
≥1	63	11.93
The distance from village to township(km) (Mean, SD) [range]	6.78,7.57 [0–115]	
The distance from village to county (km) (Mean, SD) [range]	29.86,23.35 [1–147]	

a 12 cases were missing.

b SD, standard deviation.

c HBD, home-based delivery; VLD, village-level delivery; TLD, township-level delivery; CLD, county-level-or-above delivery.

d The information of nine villages was missing.

### Care-seeking pattern for childhood diarrhea

Most of the caretakers (91.25%) reported that they had sought care for the recent diarrheal episode of their children. Slightly over one-third (36.83%) of the caretakers sought the high-level care for their children with diarrhea, including TLC (27.31%) and CLC (9.52%), and about 40% sought village-level care. About 15% took self-care of their children with diarrhea at home. Yet, 8.75% never sought any care for their children (Table 1).

**Figure 4:**
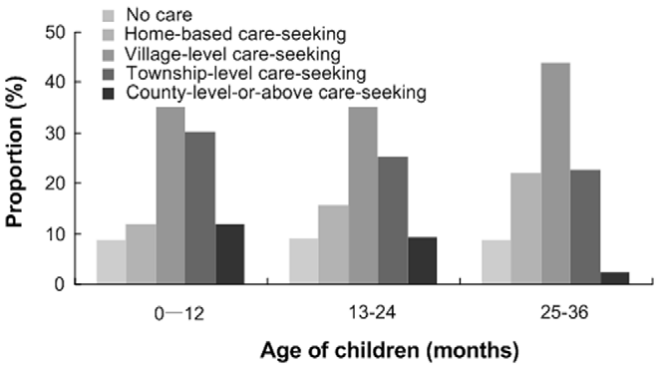
The age-specific care-seeking pattern for diarrhea among children under 36 months old.

**Figure 5:**
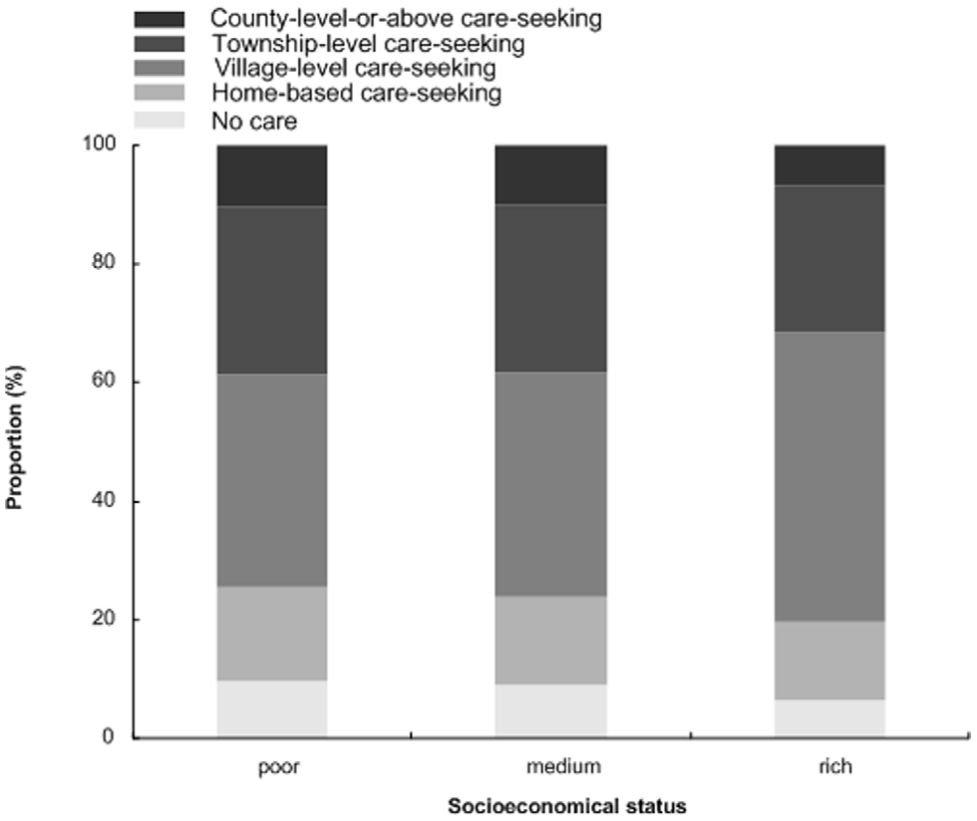
The care-seeking pattern for diarrhea in different socio-economical statuses among children under 36 months old.

In the ten investigated provinces, the overall proportion of care-seeking (HBC, VLC, TLC and CLC) was different (*x* ^2^ = 26.11, p<0.001). The proportion of VLC was significantly higher than that of HBC and CLC (VLC vs HBC: p<0.001; VLC vs CLC: p<0.001), and the proportion of TLC was also higher than that of CLC (p = 0.04). Figure 4 showed the age-specific care-seeking pattern in 3 age groups. A statistically significant difference among 4 categories of care-seeking in the 3 age groups was observed (*x* ^2^ = 10.20, p = 0.017). Of the six pairs of comparisons by Nemenyi test, there was statistically significant difference only between VLC and CLC (p = 0.029). Figure 5 displayed care-seeking pattern in the different socio-economical statuses of the households. In the 3 socio-economical statuses, the allover difference between the proportions of care-seeking was significant statistically (*x* ^2^ = 10.42, p = 0.015). Furthermore, the results of the pairwise comparisons showed that the difference also only between VLC and CLC was significant (p = 0.025).

### Determinants of care-seeking pattern

Table 3 showed the results from the GEE model analysis of care-seeking as the outcome variable among children under 36 months old. The results demonstrated that compared with the caretakers of the children delivered at county-level or above hospitals, those of the children delivered at home (−0.23, 95%CI: −0.45,−0.01, p = 0.040) seldom sought a higher level care; that the age of the children was negatively associated with seeking a higher level care (12 vs 36 months: 0.35, 95%CI: 0.16,0.55, p<0.001; 24 vs 36 months: 0.26, 95%CI: 0.08,0.44, p = 0.004); that the more danger signs of diarrhea the caretakers recognized, the higher level care they sought for their children with diarrhea (0.04, 95%CI: 0.00,0.07, p = 0.037); that the children with breastfeeding were given a higher level care than those without (0.15, 95%CI: 0.01,0.28, p = 0.035); that the mothers with the higher education sought a higher level care for their children with diarrhea than those with only primary education (0.29, 95%CI: 0.03,0.56, p = 0.032); and that the farther the villages where these caretakers lived were from their townships, the lower level care they sought for their children with diarrhea (−0.09, 95%CI: −0.18,−0.01, p = 0.039).

**Table 3:** Predictors of care-seeking pattern for diarrhea among children under 36 months old in 10 provinces of rural western China.

aVariables	Univariate			Multivariate		
	Coefficient	95%CI	p-value	Coefficient	95%CI	p-value
Province						
Gansu	−0.06	−0.53,0.41	0.805	−0.15	−0.68,0.38	0.580
Guizhou	−0.17	−0.58,0.24	0.412	−0.05	−0.51,0.40	0.819
Qinghai	−0.22	−0.63,0.19	0.296	−0.24	−0.69,0.21	0.302
Jiangxi	−0.10	−0.57,0.37	0.670	−0.16	−0.66,0.34	0.534
Xinjiang	−0.34	−0.73,0.05	0.091	−0.26	−0.72,0.20	0.261
Guangxi	0.04	−0.42,0.49	0.869	0.04	−0.47,0.54	0.889
Sichuan	0.03	−0.37,0.44	0.873	0.10	−0.35,0.54	0.669
Ningxia	0.10	−0.37,0.56	0.687	0.01	−0.49,0.50	0.977
Innter Mogolia	0.07	−0.42,0.55	0.792	0.02	−0.53,0.50	0.955
Chongqing	0.00			0.00		
Family size(≥5 persons)	−0.00	−0.16,0.15	0.973	0.03	−0.11,0.16	0.713
Pre-school-aged child size (one person)	−0.09	−0.20,0.03	0.141	0.01	−0.14,0.17	0.864
Drinking boiled water often(not)	0.13	−0.05,0.31	0.169	−0.03	−0.22,0.16	0.775
Socio-economical status						
Poor	0.01	−013,0.15	0.911	0.08	−0.08,0.24	0.329
Medium	0.05	−0.13,0.23	0.588	0.11	−0.07,0.29	0.214
Rich	0.00			0.00		
Main caretakers (others)	−0.01	−0.17,0.16	0.942	0.09	−0.09,0.26	0.341
Ethnicity (minority)	0.27	0.13,0.41	<0.001	0.16	−0.02,0.34	0.083
Maternal age	−0.01	−0.02,0.01	0.487	−0.01	−0.03,0.01	0.281
Maternal education (0–9 years)	0.34	0.07,0.61	0.013	0.29	0.03,0.56	0.032
Receiving educational materials(not)	0.14	−0.03,0.32	0.109	0.07	−0.11,0.25	0.435
Recognition of danger signs	0.03	0.00, 0.07	0.043	0.04	0.00,0.07	0.037
Age of children						
0–12 months	0.36	0.20, 0.53	<0.001	0.35	0.16,0.55	<0.001
13–24 months	0.24	0.07, 0.42	0.006	0.26	0.08,0.44	0.004
25–36 months	0.00	.	.	0.00	.	.
bThe delivery						
HBD	−0.43	−0.62, −0.24	<0.001	−0.23	−0.45,−0.01	0.040
VLD	0.09	−0.22, 0.39	0.569	0.26	−0.07,0.60	0.121
TLD	−0.05	−0.19, 0.10	0.516	−0.01	−0.15,0.14	0.933
CLD	0.00	.	.	0.00	.	.
Gender of the child (boy)	−0.07	−0.20,0.07	0.325	−0.06	−0.20,0.07	0.356
Being breastfed when surveyed (not)	0.14	0.01,0.27	0.030	0.15	0.01,0.28	0.035
Oral vitamin A (non-use)	−0.03	−0.17,0.11	0.710	0.10	−0.07,0.26	0.250
The number of retail pharmacy (zero)	0.08	−0.14,0.25	0.571	−0.02	−0.21,0.18	0.846
Distance(10 km) from village to township	−0.09	−0.17,−0.01	0.035	−0.09	−0.18,−0.01	0.039
Distance(10 km) from village to county	0.00	−0.03,0.04	0.786	−0.00	−0.03,0.03	0.962

a The items in the parentheses after the variable were used as the reference;

b HBD, home-based delivery; VLD, village-level delivery; TLD, township-level delivery; CLD, county-level-or-above delivery;

CI: confidence interval.

## Discussion

In rural western China, there are not good medical or public health services. Most of the villages have no pharmacies and village clinics are the main source of drug supplies. [18]Therefore, it is particularly critical for sick children to seek prompt and appropriate care as early as possible. Despite this, 8.75% of the diarrhea children had not received any care at all.

In fact, most cases of childhood diarrhea can be treated at home by increasing fluid intake and continuing feeding during diarrheal episodes. [5] Many studies also showed that this type of care was common for childhood diseases. [3], [9], [19]–[21] In our study, only 14.71% of children with diarrhea accepted home-based care, which was just caretakers' initial behavior of care-seeking. If caretakers could use fluids available at home to correct the dehydration when some mild symptoms appeared in children, not only severe cases but also risk of mortality could be reduced greatly. Therefore, the health intervention must emphasize early and appropriate use of rehydration fluids in home management of diarrhea. [21] Our study found that compared with other sought cares, the village-level care was utilized by the largest proportion of the children with diarrhea, as found by one previous study of the children under 5 years old in 3 provinces of China. [22] However, the village doctors, of whom more than one-third had no full-time medical education, were more inclined to adopt irrational drug utilizations. [23], [24] So, appropriate educational projects of promoting regular management for childhood diarrhea in village-level health-care settings should be implemented urgently. [23] Township-level care was the second to village-level one. It covered 27.31% of the children with diarrhea. So its role should be emphasized in the allocation of health resources for the management and treatment of childhood diarrhea in these areas.

The GEE model analysis found that the farther the villages where these caretakers lived were from their townships, the lower level care they sought for their children with diarrhea. Some studies had showed a clear impact of distance on the utilization of health care facilities. [25], [26]The longer distance to the townships not only makes the transportation from villages to townships very expensive but also makes care-seeking in townships inconvenient and inaccessible, which may weaken the caretakers' belief in seeking township-level care even if the severe symptoms appear in their children with diarrhea. So, local health care system should establish special support mechanism of care service for the households whose children are suffering from the diarrhea in those villages very far from townships. More governmental efforts should also be made to help the handy low-level medical sectors in those villages to improve their ability of medical service. Our study also found that compared with the caretakers of the children delivered in county-level or above hospitals, those of the children delivered at home seldom sought a higher level care. These mothers who had to choose to deliver their children at home possibly because they lived very far from higher level health facilities and could not afford to get there very easily or because they could not afford the care also chose to treat their children with diarrhea at home or at low-level care facilities nearby, even if when the diarrhea was quite serious. In addition, the successful delivery experience at home may also make these caretakers quite self-confident of curing their children with diarrhea at home. Some specialized health care counsels for these mothers who delivered or will deliver their children at home should be done urgently to guide them to seek appropriate care in future diarrhea episodes of their children, especially when severe symptoms appear in their children. Information of the children's birth settings is also very important for determining the priority of care-seeking intervention or care counsel. Additionally, our study found that the caretakers capable of recognizing the more danger signs of diarrhea often sought a higher level care than those who could recognize none. So, early detection of danger signs in diarrhea should be included in the self-care system. [12]In our study, merely 29% of the caretakers could recognize more than three danger signs. Hence, the programs to raise the public health awareness should be launched to help the caretakers understand the disease process and the difference between favorable and unfavorable health practices. [12]This would enhance the caretakers' capacity of understanding the disease process, the severity of the disease and the importance of preventive measures for a better family health. [12], [27] In addition, our study found that the mothers with a higher education sought a higher level care for their children with diarrhea than those with only a primary education. Highly educated mothers can utilize health information and services better and are equipped better for initiating and controlling decision making with regard to health. [28], [29] Mothers' higher levels of education can also help increase their ability to recognize the diarrhea. On the other hand, with more and more people with higher levels of education living in or close to urban areas, a high level health facility is easily available to access, so they are more likely to use that facility rather than treat their children with diarrhea at home or nearby low level health care center. Besides, our study found that the caretakers whose children were aged 0–12 months and 13–24 months sought the higher level care than those whose children were aged 25–36 months in the diarrheal episodes. The recent studies have also showed the age of children had the similar association with the utilization of health facility. [8], [29] Perhaps childhood physiology makes sense. Younger children have less “reserve”- their physiology tolerates less diarrhea and they can get dehydrated more easily. Older children with diarrhea are less likely to have severe cases and therefore have less need for higher level care. Our study also found that the children with breastfeeding were given the higher level care than those without. The possible reason was that the children during lactation were generally of smaller age or that those mothers who insist on breastfeeding their children may be highly alert to the health of their children, especially when some symptoms appear. So emphasis on correct care concept about childhood diarrhea should be made to those mothers whose children with diarrhea are not being breastfed or beyond lactation.

Several limitations in the current study should be acknowledged. All data were collected on the basis of caretakers' recall, so the estimated results of care-seeking are subject to recall bias. Some potential factors such as the time of care-seeking, the perceived severity of diarrhea, the frequency of diarrheal episodes in the previous two weeks and the expected cost of the care by perceived status of diarrhea and so on, which had not been collected in the current study, may confound the findings of this study.

In conclusion, village-level and township-level care were sought for childhood diarrhea by most of the caretakers. Birth settings, the distance from village to township, maternal education, caretakers' awareness of the danger signs of diarrhea, breastfeeding status and age of children affected the care-seeking. These findings may have some implications for the improvement of health care services and care-seeking intervention against childhood diarrhea in rural western China.
